# Long-term follow-up of tonsillectomy efficacy in children with PFAPA syndrome^☆^

**DOI:** 10.1016/j.bjorl.2017.10.012

**Published:** 2017-11-21

**Authors:** Ozturk Aktas, Hande Gurbuz Aytuluk, Sebla Kumas Caliskan, Omer Erdur, Ahmet Adnan Cirik

**Affiliations:** a Kocaeli State Hospital, Department of Otolaryngology, Kocaeli, Turkey; b Kocaeli State Hospital, Department of Anesthesiology and Reanimation, Kocaeli, Turkey; c Derince Training and Research Hospital, Department of Otolaryngology, Kocaeli, Turkey; d Selcuk University, Department of Otolaryngology, Konya, Turkey; e Umraniye Training and Research Hospital, Department of Otolaryngology, Istanbul, Turkey

**Keywords:** Fever, Lymphadenitis, Stomatitis aphthous, Pharyngitis, Tonsillectomy, Febre, Linfadenite, Estomatite aftosa, Faringite, Tonsilectomia

## Abstract

**Introduction:**

The role of tonsillectomy in the periodic fever, aphthous stomatitis, pharyngitis, and adenitis syndrome, is controversial. Although some studies reported high success rates with tonsillectomy, further investigations are needed with larger numbers of patients.

**Objective:**

To seek the long-term outcomes of tonsillectomy in periodic fever, aphthous stomatitis, pharyngitis, and adenitis syndrome.

**Methods:**

Case series; multi-center study. The study comprised 23 patients with periodic fever, aphthous stomatitis, pharyngitis, and adenitis syndrome who underwent surgery (tonsillectomy with or without adenoidectomy) between January 2009 and November 2014.

**Results:**

21 (91%) of 23 patients had complete resolution immediately after surgery. One patient had an attack 24 h after surgery, but has had no further attacks. One patient had three attacks with various intervals, and complete remission was observed after 3 months.

**Conclusions:**

Tonsillectomy is a good option for the treatment of periodic fever, aphthous stomatitis, pharyngitis, and adenitis syndrome.

## Resumo

**Introdução:**

O papel da tonsilectomia na síndrome da febre periódica, estomatite aftosa, faringite e adenite é controverso. Embora alguns estudos tenham relatado altas taxas de sucesso com a tonsilectomia, são necessárias mais pesquisas com um número maior de pacientes.

**Objetivo:**

Avaliar os resultados em longo prazo da tonsilectomia na síndrome de febre periódica, estomatite aftosa, faringite e adenite.

**Método:**

Série de casos; estudo multicêntrico. O estudo avaliou 23 pacientes com síndrome de febre periódica, estomatite aftosa, faringite e adenite submetidos a cirurgia (tonsilectomia com ou sem adenoidectomia) entre janeiro de 2009 e novembro de 2014.

**Resultados:**

Dos 23 pacientes, 21 (91%) apresentaram resolução completa imediatamente após a cirurgia. Um paciente apresentou um episódio 24 horas após a cirurgia, mas sem recorrência posterior. Um paciente teve três episódios com vários intervalos e a remissão completa foi observada após 3 meses.

**Conclusões:**

A tonsilectomia é uma boa opção para o tratamento da síndrome de febre periódica, estomatite aftosa, faringite e adenite.

## Introduction

The Periodic Fever, Aphthous Stomatitis, Pharyngitis, and Cervical Adenitis (PFAPA) syndrome was first described by Marshall et al. in 1987^1^; however, its cause is still unknown. It usually begins before the age of 5 years, and ends pre-puberty. The main symptom of this syndrome is episodes of fever that last for 3–6 days with recurrence every 3–8 weeks. One or more of following symptoms can be seen during an attack: aphthous stomatitis, pharyngitis, and cervical adenopathy. Rarely, patients experience rashes, headaches, abdominal pain or arthralgia. Patients are asymptomatic between episodes and show normal growth and development.

The role of tonsillectomy in PFAPA syndrome is controversial. Although some studies reported high success rates with tonsillectomy, further investigations are needed with larger numbers of patients. In this study, we report the efficacy of tonsillectomy in PFAPA syndrome in 23 children.

## Material and methods

Twenty-three patients with PFAPA syndrome who underwent surgery (tonsillectomy with or without adenoidectomy) at three different hospitals (Kocaeli State Hospital, Golcuk Government Hospital, and Derince Training and Research Hospital) in Kocaeli, between January 2009 and November 2014, were identified. All patients were diagnosed according to PFAPA criteria established by Thomas and colleagues.^2^ Each patient had regularly recurring fevers with an early age of onset (less than five years of age), symptoms in the absence of upper respiratory tract infection, with at least one of aphthous stomatitis, cervical lymphadenitis, and pharyngitis, completely asymptomatic intervals between episodes, and normal growth and development. All of 23 patients were monitored during each episode for 6 months before surgery, and followed up once a month at least for 12 months after surgery. Twenty-one patients underwent adenotonsillectomy and two patients underwent tonsillectomy without adenoidectomy because of the absence of obstructive symptoms and clinical findings. Preoperative complete blood cell counts were obtained during all febrile episodes to exclude cyclic neutropenia. No abnormal changes were observed in the routine biochemistry analyses. All of the patients’ vitamin D levels were within normal range. Patients were referred to the pediatric clinic before surgery.

The study was approved by the Kocaeli University Ethics Committee of Noninvasive Investigations (26.04.2017; protocol no. 2017/130; decree no. 2017/6.25) and the parents of each child included in the study were informed about the aim of the study and asked for written informed consent before inclusion in the study.

## Results

Twenty-three patients (14 males, 9 females) with PFAPA syndrome, aged between 36 months and 84 months were included in the study. The average age of symptom onset was 27 months (range, 12–36 months). Episodes recurred every 3–8 weeks (mean: 3.9 weeks). During episodes, fever was observed for a mean of 3.7 days. Pharyngitis (18/23) was the most common clinical manifestation. Cervical adenitis (14/23) and aphthous stomatitis (10/23) were also observed. The mean age at the time of surgery (tonsillectomy with or without adenoidectomy) was 50 months (range, 36–84 months). No major complications were observed after surgery. All patients completed the study. Of the 23 patients, 21 had complete symptom resolution immediately after surgery. Only two patients did not have resolution of fevers after surgery. One patient had an attack 24 h after surgery, but has had no further attacks (case 11). One patient had three attacks with various intervals, and complete remission was observed after three months (case 18). The demographics and clinical characteristics of the patients are presented in Table 1, Table 2.

**Table 1:** Preoperative demographic and clinical characteristics of PFAPA patients.

Characteristic	PFAPA patients (*n* = 23)
Male; *n* (%)	14 (61%)
Female; *n* (%)	9 (39%)
Age at onset; mean (range), months	27 (12–36)
Recurrence of episodes; mean (range), weeks	3.9 (3–8)
Duration of fever; mean (range), days	3.7 (3–5)
Pharyngitis; *n* (%)	18 (78%)
Cervical adenitis; *n* (%)	14 (61%)
Aphthous stomatitis; *n* (%)	10 (43%)
Age at surgery; mean (range), months	50 (36–84)

**Table 2:** Clinical and surgical presentation.

Case no.	1	2	3	4	5	6	7	8	9	10	11	12	13	14	15	16	17	18	19	20	21	22	23
Male/female	M	F	M	M	M	M	M	F	M	F	F	M	F	M	M	M	F	F	F	M	M	M	F
Age onset, months	12	12	13	14	14	18	22	24	24	28	28	32	32	32	33	34	34	35	36	36	36	36	36
Recurrence of episodes, weeks	3	8	3	3	3	6	3	4	3	4	4	4	3	5	3	3	4	3	3	4	3	6	4
Duration of fever, days	3	3	4	3	3	5	5	3	4	3	3	5	3	3	5	3	5	3	3	3	5	5	3
Pharyngitis	−	+	−	−	+	+	+	−	+	+	+	+	+	+	+	−	+	+	+	+	+	+	+
Aphthous stomatitis	+	+	+	+	−	−	−	−	+	+	−	−	+	−	−	−	+	+	−	−	−	+	−
Cervical adenitis	−	−	−	+	−	+	+	−	−	+	+	−	+	−	+	+	−	+	+	+	+	+	+
AT/T	AT	AT	AT	AT	AT	AT	AT	AT	AT	AT	AT	AT	T	AT	AT	AT	AT	AT	AT	T	AT	AT	AT
Age at surgery, months	48	40	55	44	36	36	39	36	36	41	37	38	52	84	72	50	40	80	42	60	68	70	46

Cases are ordered according to their ages.

AT, adenotonsillectomy; T, tonsillectomy.

## Discussion

PFAPA syndrome can be diagnosed by the exclusion of other causes of regular, repeated episodes of fever, such as cyclic neutropenia, Familial Mediterranean Fever (FMF), hyperglobulinemia D syndrome, Behcet's disease, juvenile rheumatoid arthritis, and autosomal dominant Hereditary Periodic Fever syndrome (HPF).^2^, ^3^ We believe that the most important criterion of studies about PFAPA syndrome must be patient selection. Accordingly, we paid great attention to patient selection in our study. In most studies, we see that many patients do not meet all PFAPA syndrome criteria. In contrast, all diagnoses in the present study were made in accordance with the PFAPA criteria established by Thomas et al.^2^ Complete blood cell counts were obtained during all febrile episodes to exclude cyclic neutropenia. Throat cultures were negative for all patients during attacks. Patients were referred to the pediatric clinic to exclude other causes.

The unknown pathogenesis of PFAPA syndrome causes uncertainty in its treatment. There are few studies about the treatment of this syndrome. The suggested treatments consist of conservative (pharmacologic) therapies and surgical intervention. Treatment with corticosteroids appears to be the most effective nonsurgical therapy. Prednisone (1–2 mg/kg) or betamethasone (0.1–0.2 mg/kg) are effective agents for aborting fever attacks within a few hours.^4^, ^5^, ^6^ However, other symptoms can take longer to resolve. No systemic toxicity or adverse effects related with these doses of corticosteroids have been reported. The disadvantage of corticosteroid therapy is that it does not prevent future fever attacks and can even shorten the interval between attacks.^4^, ^7^, ^8^, ^9^, ^10^ Steroid response may be useful in distinguishing PFAPA episodes from other differential diagnoses such as FMF or HPF,^5^, ^11^ and can be used for additional diagnostic criteria.^12^ Despite the fact that steroids are the most effective drugs for the treatment of symptoms, we do not believe that they are a good option for long-term use because they do not solve the root of the problem or prolong intervals between febrile episodes.

Colchicine is a good drug for reducing inflammation. Oral colchicine 0.5–1 mg per day may reduce fever frequency. Like steroids, colchicine does not provide complete remission.^13^ The rationale for the use of colchicine in PFAPA prophylaxis is based on clinical and laboratory similarities between FMF and PFAPA. Accordingly, if colchicine is effective in a patient with PFAPA, a differential diagnosis of FMF has to be considered.^4^, ^13^, ^14^, ^15^

Cimetidine has immune-modulating effects through the inhibition of chemotaxis and T-cell activation. Oral cimetidine 20–40 mg/kg per day can also be used for prophylaxis.^2^, ^16^ However studies showed that cimetidine therapy does not promise much hope.^5^, ^13^, ^17^, ^18^

Interleukin-1 plays a key role in PFAPA pathogenesis. In a small sample, it was shown that a single subcutaneous injection of anakinra dramatically improved both the clinical picture and laboratory parameters.^6^, ^19^, ^20^ At this point, further investigations are needed with larger numbers of patients.

In addition to the findings that vitamin D levels are associated with inflammatory disorders, vitamin D is considered to be a possible regulator of inflammation.^21^ Stagi et al. and Mahamid et al. found a significant correlation between PFAPA and vitamin D deficiency in their studies; a significant reduction in the frequency of febrile episodes was observed in patients after vitamin D supplementation.^22^, ^23^ In spite of these limited data, it is not possible to conclude that vitamin D is effective in PFAPA syndrome.

The role of surgery in the treatment of PFAPA syndrome is still controversial. Although PFAPA syndrome is a self-limiting disease, tonsillectomy with or without adenoidectomy as a surgical procedure, seems to be a good option for treating PFAPA. Several studies previously reported high success rates with tonsillectomy. Contrarily, a long-term observational study that compares efficacy of tonsillectomy and medical treatment (prednisone and non-steroidal anti-inflammatory drugs) showed no significant difference between the two methods.^24^ Unfortunately, unpredictable but finite periods of recurrent episodes at predictable intervals require time off school and being prescribed regular medication during this period can be very traumatic for patients and parents.^25^

Abramson was the first author to report the efficacy of tonsillectomy in four children with PFAPA in 1989.^26^ In 2000, a retrospective study was conducted by Dahn et al. that included five patients who underwent tonsillectomy and none had any attacks after surgery.^27^ Another study including 15 patients by Galanakis et al. showed 100% success after tonsillectomy.^3^ Afterwards, a randomized controlled trial that compared 14 patients who underwent tonsillectomy and 12 non-surgical control patients was conducted by Renko et al.^28^ The syndrome resolved immediately in all 14 patients who underwent surgery; in contrast, the syndrome resolved spontaneously within 6 months in 6 patients who had no surgery. However, a weakness of their study was that most patients did not actually fulfill the PFAPA criteria. In a retrospective analysis of 9 patients who underwent tonsillectomy by Wong et al., complete remission was achieved immediately in 8 patients, and the frequency of attacks were decreased in the patient who did not achieve immediate remission.^29^ In Garavello et al.’s prospective randomized controlled trial, 39 patients with PFAPA were included.^30^ Nineteen patients underwent adenotonsillectomy and 20 were treated with medical therapy. After 18 months’ post-surgical follow-up, the authors observed complete resolution in all patients who underwent surgery; only one patient in the control group showed spontaneous resolution. Pignataro et al. performed a randomized controlled trial,^31^ including 18 patients with PFAPA, who were divided into two groups; 9 surgical patients and 9 non-surgical patients. All of the nine surgical patients showed symptomatic improvement, with complete clinical recovery in 5 patients, and a significant reduction of frequency and duration of fever episodes in the remaining four. Of the 9 patients in the non-surgery group, eight had continued relapse and remission periods, and one of these patients was scheduled for surgery. The ninth patient was lost to follow-up. Licameli et al. demonstrated complete symptom cessation in 26 of 27 patients after surgery in a prospective study in 2008; the child who continued to have febrile episodes had tumultuous intervals.^8^ Another prospective study by Licamelli et al. evaluated the long-term efficacy of adenotonsillectomy in 102 patients with a wide range of ages (18 months to 18 years) in 2012.^32^ Ninety-nine patients had complete resolution immediately after surgery, and one patient achieved resolution six months after surgery. Of the remaining two patients, one continued to have episodes and the other was further investigated and diagnosed as having mevalonate kinase deficiency.

We think that our study shows that surgery is an effective treatment option for PFAPA syndrome. Twenty-one (91%) of 23 patients had complete resolution immediately after surgery. One patient had an attack 24 h after surgery, but has had no further attacks. It is possible that the patient underwent surgery at the overlapping time of a subclinical attack. One patient had three attacks with various intervals, but achieved a complete remission after 3 months. Our study is limited by not having a control group for comparison.

## Conclusion

PFAPA resolves spontaneously and treatment can be administered to try to reduce the severity of individual episodes. Pharmacological therapies reduce attack duration but do not prevent future fever attacks. A second option is tonsillectomy. Tonsillectomy is, however, an invasive treatment and the child's parents must weigh the risks and consequences of surgery. The high success rate of future fever attack prevention shows us that tonsillectomy (with or without adenoidectomy) is a good alternative for the treatment of PFAPA.

## Conflicts of interest

The authors declare no conflicts of interest.
